# Supplementary figures and images for: Effects of changes in isotopic baselines on the evaluation of food web structure using isotopic functional indices
Source: PeerJ. 2020 Oct 26;8:e9999. doi: 10.7717/peerj.9999 (PMC7594636; doi:10.7717/peerj.9999)

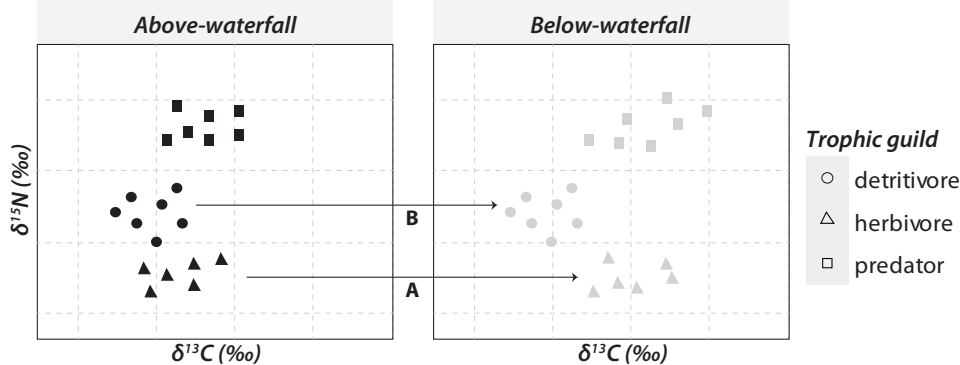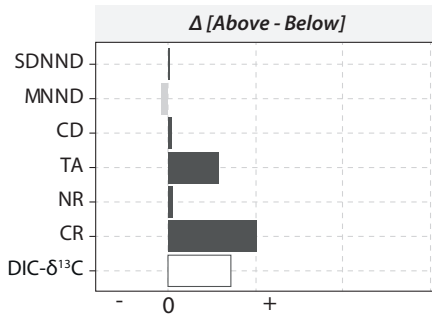

Supplement: Supplemental Information 1 [file peerj-08-9999-s001.pdf]
