# Supplementary material for: Effects of changes in isotopic baselines on the evaluation of food web structure using isotopic functional indices
Source: PeerJ. 2020 Oct 26;8:e9999. doi: 10.7717/peerj.9999 (PMC7594636; doi:10.7717/peerj.9999)
Supplement: Supplemental Information 3 [file peerj-08-9999-s003.docx]

| **Period** | **Location** | **Proxy** | **Value** |
| --- | --- | --- | --- |
| Early_May | Above | pCO2_SR | 1.16 |
| Early_May | Above | pCO2_SR | 1.28 |
| Early_May | Above | pCO2_SR | 3.8 |
| Early_May | Above | pCO2_SR | 4.84 |
| Early_May | Above | pCO2_SR | 0.77 |
| Early_May | Above | pCO2_SR | 1.07 |
| Early_May | Below | pCO2_SR | 0.79 |
| Early_May | Below | pCO2_SR | 0.83 |
| Early_May | Below | pCO2_SR | 0.85 |
| Early_May | Below | pCO2_SR | 0.6 |
| Early_May | Below | pCO2_SR | 0.96 |
| Late_June | Above | pCO2_SR | 0.96 |
| Late_June | Above | pCO2_SR | 1.16 |
| Late_June | Above | pCO2_SR | 2.48 |
| Late_June | Above | pCO2_SR | 1 |
| Late_June | Above | pCO2_SR | 0.97 |
| Late_June | Above | pCO2_SR | 0.95 |
| Late_June | Above | pCO2_SR | 1.32 |
| Late_June | Above | pCO2_SR | 0.98 |
| Late_June | Below | pCO2_SR | 0.94 |
| Late_June | Below | pCO2_SR | 0.94 |
| Late_June | Below | pCO2_SR | 1.08 |
| Late_June | Below | pCO2_SR | 0.4 |
| Late_June | Below | pCO2_SR | 0.3 |
| Late_June | Below | pCO2_SR | 0.72 |
| Late_June | Below | pCO2_SR | 0.74 |
| Late_June | Below | pCO2_SR | 1.06 |
| Early_May | Above | DIC | -12.04 |
| Early_May | Above | DIC | -11.67 |
| Early_May | Above | DIC | -12.85 |
| Early_May | Above | DIC | -8.28 |
| Early_May | Above | DIC | -8.31 |
| Early_May | Above | DIC | -8.5 |
| Early_May | Below | DIC | -7.99 |
| Early_May | Below | DIC | -6.96 |
| Early_May | Below | DIC | -6.27 |
| Early_May | Below | DIC | -6.59 |
| Early_May | Below | DIC | -8.64 |
| Early_May | Below | DIC | -6.41 |
| Late_June | Above | DIC | -8.28 |
| Late_June | Above | DIC | -8.18 |
| Late_June | Above | DIC | -10.97 |
| Late_June | Above | DIC | -10.65 |
| Late_June | Above | DIC | -7.54 |
| Late_June | Above | DIC | -7.15 |
| Late_June | Above | DIC | -11.98 |
| Late_June | Above | DIC | -10.54 |
| Late_June | Above | DIC | -8.77 |
| Late_June | Above | DIC | -9.09 |
| Late_June | Below | DIC | -5.39 |
| Late_June | Below | DIC | -5.18 |
| Late_June | Below | DIC | -7.04 |
| Late_June | Below | DIC | -7.04 |
| Late_June | Below | DIC | -6.39 |
| Late_June | Below | DIC | -6.42 |
| Late_June | Below | DIC | -9.55 |
| Late_June | Below | DIC | -10.07 |
| Late_June | Below | DIC | -6.63 |
| Late_June | Below | DIC | -7.02 |
| Early_May | Above | Temp | 3.97 |
| Early_May | Above | Temp | 6.68 |
| Early_May | Above | Temp | 5.68 |
| Early_May | Above | Temp | 5.44 |
| Early_May | Below | Temp | 3.65 |
| Early_May | Below | Temp | 4.97 |
| Early_May | Below | Temp | 6.53 |
| Early_May | Below | Temp | 5.58 |
| Late_June | Above | Temp | 20.74 |
| Late_June | Above | Temp | 20.32 |
| Late_June | Above | Temp | 21.39 |
| Late_June | Above | Temp | 23.1 |
| Late_June | Above | Temp | 16.7 |
| Late_June | Above | Temp | 15.99 |
| Late_June | Below | Temp | 18.47 |
| Late_June | Below | Temp | 20.2 |
| Late_June | Below | Temp | 21.73 |
| Late_June | Below | Temp | 22.32 |
| Late_June | Below | Temp | 15.8 |
| Late_June | Below | Temp | 15.8 |
